# Re‐Evaluating Access in Healthcare: Focus on Quality of Care

**DOI:** 10.1002/puh2.70100

**Published:** 2025-11-11

**Authors:** Allen M. Chen

**Affiliations:** ^1^ Department of Radiation Oncology University of California, Irvine, Chao Family Comprehensive Cancer Center Orange California USA

**Keywords:** access, barriers, health policy, inclusion, social determinants

## Abstract

The ability of patients to readily and seamlessly obtain healthcare services is a crucial element of high‐quality medical care. Access—defined by the National Academy of Medicine as “the timely use of personal health services to achieve the best health outcome”—directly impacts individual and population health as efforts to optimize access inherently promote well‐being and reduce disparities. The link between access and quality is so fundamental to the patient experience that they have become increasingly intertwined as a unified concept. However, due to the sheer complexity of the healthcare system, barriers to access are gradually being recognized as the boundaries among technology, medicine, business, public health, and policy become blurred. The contribution of many human elements, including those stemming from the provider–patient relationship, in optimizing access must also be recognized. Ultimately, improving access requires concerted engagement from a myriad of stakeholders with the goal of prioritizing timely, equitable, personalized, and high‐quality care, while empowering patients to take charge of their own health. Although it presents a profound challenge, the journey toward bridging the many gaps is just beginning, and how society re‐defines the access imperative in healthcare in an ever‐evolving landscape represents one of the foremost issues of the future. This is because access is dependent on ensuring that high‐quality, evidence‐based healthcare resources are available for everyone. Indeed, the implications for society are tremendous given that access is central to quality of care, profoundly impacts the patient experience, and influences health outcomes. The purpose of this review is to outline the core issues that contribute to this paradigm while focusing on exploring the relationships between access and quality of care.

## Graphical abstract

The feasibility of evaluating healthcare access in the context of the six domains of healthcare quality as recommended by the Institute of Medicine was demonstrated: Timeliness, Safety, Effectiveness, Efficiency, Equity, and Patient‐centered.

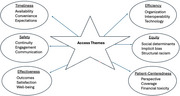

## 1 Introduction

The link between healthcare access and quality is intuitively understood. According to the World Health Organization (WHO), “the enjoyment of the highest attainable standard of health is one of the fundamental rights of every human being without distinction of race, religion, political belief, economic or social condition” [1]. However, barriers to healthcare are increasingly being recognized and serve to practically limit the receipt of timely, appropriate, and high‐competency services for many individuals. Indeed, access to care—defined by the National Academy of Medicine as “the timely use of personal health services to achieve the best health outcome”—is now commonly cited as one of the most critical determinants of quality [2]. Given that patient waits for health services are at historical highs, studies attempting to better understand the relationship between access and quality are needed. For patients, access problems propagate a feeling of depersonalization with healthcare organizations and their providers, which can lead to frustration, anxiety, and depression, thus detrimentally impacting the patient experience. Furthermore, studies from across health conditions have shown that delays in care can be devastatingly harmful, leading, in some cases, to worsening and/or progression of disease without timely diagnosis and treatment [3, 4, 5, 6, 7]. For healthcare organizations, access problems can have negative financial consequences, as the inability to usher patients into the system in a way that is efficient and timely can compromise cost‐effective care and also erode brand loyalty and trust from communities.

Despite these observations, barriers to access continue to be pervasive among populations. Because access has generally been equated in an overly simplistic manner strictly to the availability of medical services, definitions have historically centered on this concept. This viewpoint can be shortsighted, as access is actually driven by multiple disparate factors across every level of society. Indeed, it is increasingly obvious that social determinants heavily influence the provisioning of health services. Neglecting crucial factors like affordability, geographic accessibility, cultural competency, language barriers, implicit bias, and education, including health literacy, which can significantly impede individuals from actually utilizing services even when they are technically available, has the potential to undermine efforts to improve access. In this sense, the concept of healthcare access needs redefining to incorporate a broader understanding of the patient‐centered, systems‐related, and societal contributions. Given the importance of access to health systems and organizational leaders, the purpose of this review is to outline the core issues that contribute to access, focusing on practical considerations with respect to quality of care.

## 2 Methods and Materials

This study was designed and conducted by a single author (A.M.C.) based on the Preferred Reporting Items for Systematic Review and Meta‐Analysis Protocols (PRISMA‐P) statement. A comprehensive literature search of peer‐reviewed publications was undertaken to identify original peer‐reviewed works pertaining to access to healthcare services using a variety of search terms, including “access improvement,” “quality of care,” “appointments,” “timely,” and “delays.” The initial screening was conducted on February 1, 2024, and repeated again on March 1, 2024, April 1, 2024, and April 14, 2024. With the goal of critically evaluating high‐level evidence, which could enable the preparation of this review, the focus of this work was on specifically identifying original research reporting on the impacts of access on patient care and/or health outcomes. To ensure that all possible publications were captured, multiple iterations of the search were processed on different days. Boolean operators were routinely used to combine search terms, and advanced field tags were incorporated to refine the selection process in an attempt to limit the analysis to clinically oriented studies focused on healthcare access. Reference lists from included articles were cross‐checked to identify additional articles. Review articles and papers presented as conference proceedings were excluded. Articles published from January 2014 to January 2024 with full text available on MEDLINE and restricted to the English language and human subjects were included. The full bibliographies of identified articles were reviewed, and irrelevant studies, including those focused exclusively on the waiting time while physically in the office, were selectively removed. Where individual patients were included in multiple published series, the most complete or recent article was cited.

An interpretive synthesis of the evidence evaluating the fundamental role of access was undertaken using guidelines espoused by the JBI Manual for Evidence Synthesis focusing on Scoping Reviews [8]. The PCC framework (population, concept, and context) was utilized as a guide to construct clear and meaningful objectives and eligibility criteria for this review and to identify works that could be used to construct the core framework for presentation [9]. For the purpose of thematic development, basic qualitative content analysis using a descriptive approach to analysis and involving a process of open coding to allocate concepts or characteristics into overall categories was conducted [10]. Core questions for discussion focused on healthcare access were subjectively devised on the basis of the review of the relevant peer‐reviewed literature. The results of the review were then presented descriptively in the context of the 6 domains of healthcare quality as recommended by the Institute of Medicine: Timeliness, Safety, Effectiveness, Efficiency, Equity, and Patient‐centeredness.

## 3 Results

**FIGURE 1:**
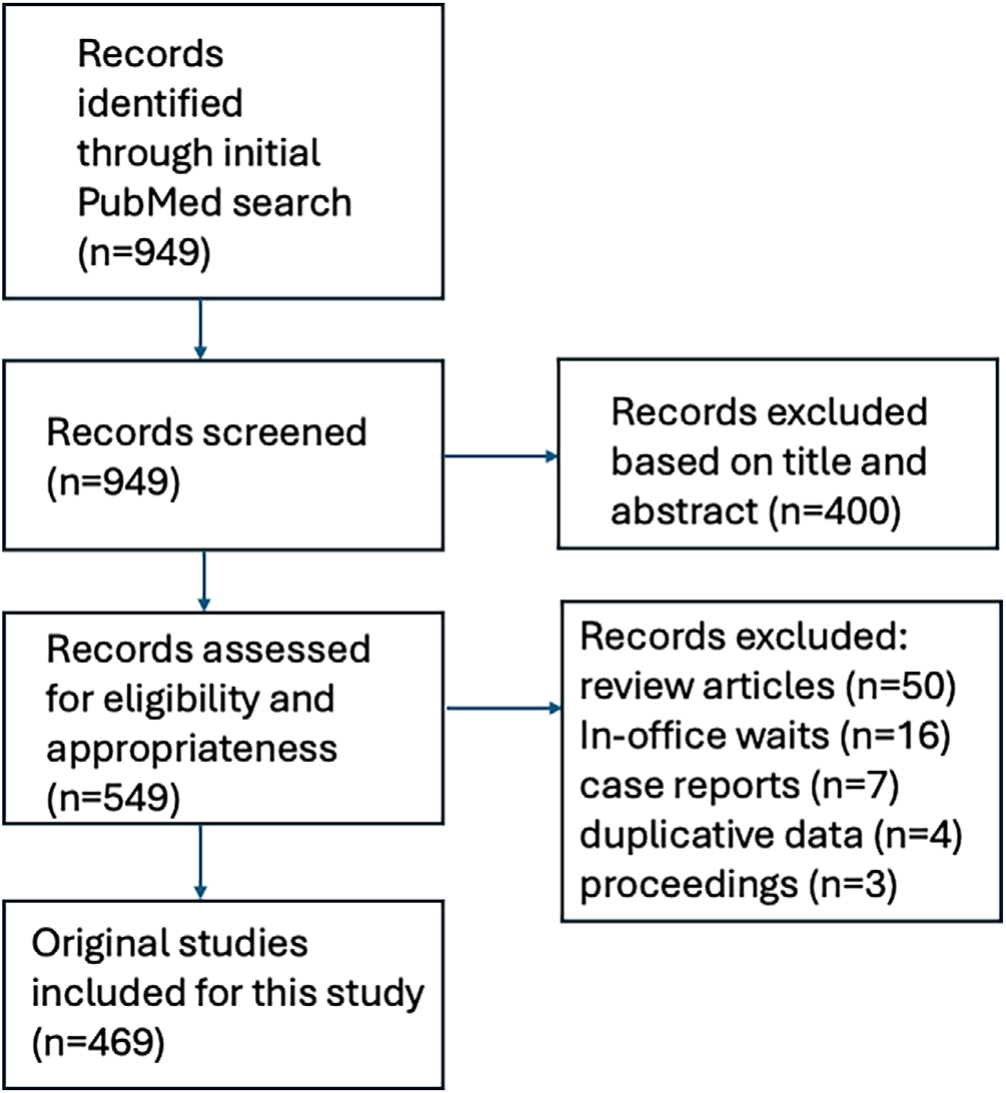
Schematic flowchart of search strategy.

The initial search yielded 949 articles. After screening these articles on title and abstract, a total of 549 studies proceeded to full‐text screening. Another 80 articles were excluded because they were review articles (*N* = 50), were more focused on in‐office delays rather than waits for providers and/or procedures (*N* = 16), were designed as narratives or case reports (*N* = 7), used duplicative data (*N* = 4), or were abstracts only or conference proceedings (*N* = 3). A total of 469 peer‐reviewed studies thus were included and formed the basis for this systematic review. Among these 469 original works, a total of 398 originated from North America (85%). A schematic illustration of the flowchart outlining the results of the search strategy is shown in Figure 1.

**FIGURE 2:**
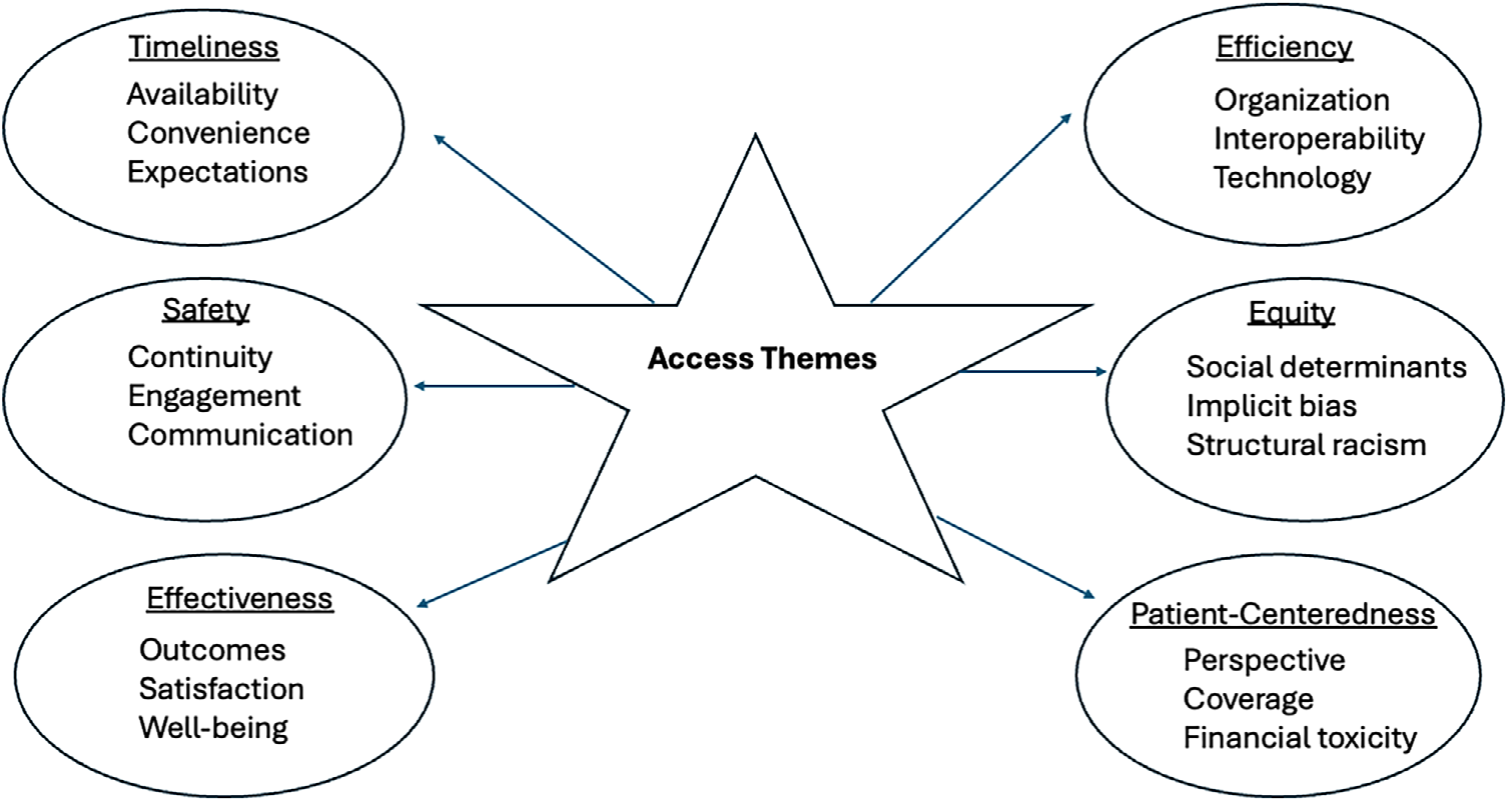
Identified access themes in relation to the six domains of healthcare quality.

The 469 studies that were identified differed significantly in their clinical design, methods, and endpoints. The most common diseases analyzed were related to general medicine/family practice (*N* = 75), surgical care (*N* = 51), health screening (*N* = 30), mental health (*N* = 27), cardiovascular disease (*N* = 17), emergency room/critical care (*N* = 15), and cancer (*N* = 7). The remaining 247 studies (53%) did not specifically report on any specialization. The sample size of human subjects ranged from 33 to 10,550 (mean, 120 patients; median, 151; standard deviation, ±95). Sixty publications (13%) focused at least in part on equity issues, structural racism, and/or implicit bias; a total of 25 publications (5%) addressed disparities in education, training, and/or technical literacy. Seventy‐three publications (16%) focused either completely or in part on digital health as a means of access improvement. Figure 2 outlines the core elements that were identified through the process of thematic development in relation to how they aligned with the six domains of healthcare quality: Timeliness, Safety, Effectiveness, Efficiency, Equity, and Patient‐centered.

## 4 Discussion

Through this evidence‐based exercise, it was possible to identify broad categorical themes that emerged to develop a framework for exploring the association between healthcare access and quality. It must be recognized that because priorities around access can be considered subjective, discretion was utilized to select the articles included and to categorize them into the theme‐based groups. Relatedly, this study did not present any quantitative data but instead only offered an interpretive synthesis of the available literature in qualitative fashion. As such, one of the primary limitations of this study was that the presented framework was devised as an interpretive construct of the author and subject to unforeseen biases. Given that the overwhelming proportion of studies included in this review originated from North America, it is also important to recognize that this framework for interpreting the relationship between access and quality may lack generalizability across other regions. For instance, less developed parts of the world may be affected by unique barriers related to infrastructure or resources that were not discussed. Nonetheless, the results presented herein may offer an instructive perspective on how to view the relationship between healthcare access and quality of care through the following domains.

### 4.1 Timeliness

The most visible definition of access centers around the availability of appointments for patients with the ostensible goal of expediting care. However, current estimates suggest that approximately 15% of adults in the United States cannot access healthcare in a reasonably rapid fashion [11]. How “reasonably” is interpreted in this context, however, remains uncertain. For instance, what might be appropriate for one individual might not be for another depending on the circumstance. What is becoming increasingly evident, however, is that patients are prioritizing access like never before. McKinsey and Company surveyed nearly 3000 healthcare consumers on which criteria mattered to them when choosing a primary care provider. Out of 20 options, respondents consistently identified “appointment availability” and “appointment times that meet your needs” as among the top factors [12]. Additional data from Accenture demonstrated that nearly two in three consumers would switch healthcare providers for the ability to obtain an appointment quickly when needed [13]. Similarly, Kleij et al. conducted a systematic review of discrete choice experiments analyzing patient preferences to identify factors that could make healthcare delivery more responsive in the primary care setting [14]. Among the 18 studies, the authors found that the most commonly applied structure attribute was “waiting time” for appointment.

Furthermore, expectations for timeliness have been shown to vary among patients and providers, suggesting that a disconnect might influence satisfaction in certain circumstances. Unsurprisingly, studies have also suggested that a gap between perception and reality might exist with respect to how patients and providers value access [15, 16, 17]. Barry et al. showed that even when physicians think that they are providing timely service, this sentiment, in fact, might not be shared by patients [15]. In a survey study conducted at two community‐based, ambulatory internal medicine clinics, the authors showed that patients expected to be seen sooner than physicians thought necessary for many common chronic medical conditions. How to reconcile these mismatched perceptions between patients and providers needs further study. However, given that evidence has shown that delays in care can compromise the efficacy of treatment, the desires of patients to be seen earlier are understandable. Although factors such as acuity and complexity of care certainly contribute to feelings of what an acceptable wait might be for an appointment, it is notable that a relative dearth of standards exists to promote timeliness. Moreover, data have increasingly suggested that simply having availability is not enough, as the lack of convenient openings could hinder access for patients for whose jobs and/or transportation considerations limit access [18]. In this sense, it is crucial to understand patient expectations for access and to consider their individual needs and priorities. Although concierge care has been developed to offer a high degree of personalization with access, these services are generally only available to a small segment of the population due to their expense. Clearly, initiatives that are designed for the masses and that consider the logistics of access are needed if improvements in timely care are to occur.

### 4.2 Safety

The link between access and the provisioning of safe care has increasingly been explored. Intuitively, delays in care can lead to worsening of disease, which results in increased hospitalizations and/or procedures that might not have been necessary if timely services had been rendered in the first place [3, 4, 5, 6, 7]. These delays can also lead to complications necessitating more intensive care. Furthermore, studies have shown that a considerable proportion of visits to the emergency room, a cost‐ and resource‐intensive use of services, could have been avoided with more routine use of primary care for low‐acuity needs [19]. The perception held among many patients that it is easier and/or more convenient to obtain care in the emergency room than in the outpatient setting is pervasive and leads to waste [20, 21].

Additionally, access problems invariably lead to rushed and fragmented care as providers scramble to make up time after delays. As studies have shown, hurrying in this manner can result in shortcuts and/or mishaps—even systemically creating an environment where accidents are more likely to occur [22, 23, 24]. For instance, unsafe medication practices and errors—such as incorrect dosages or infusions, unclear instructions, and inappropriate prescriptions—are a leading cause of avoidable harm in healthcare worldwide and have been shown to occur when providers are hurried [25]. If anything, access helps create a culture where safety is prioritized by promoting the methodical and consistent delivery of care.

The negative effect of access delays has also been dramatically shown with respect to the decreased utilization of preventive care [26]. For instance, during the COVID‐19 pandemic, cancer screenings were postponed, resulting in many patients missing their regularly scheduled mammograms or colonoscopies [27]. As a result, data have suggested an enormous excess of preventable deaths will occur over the next decade because of pandemic‐related delays in diagnosis and treatment of breast and colorectal cancer alone [28]. Outside of oncology, delays in preventive services with respect to cardiovascular, neurological, and pulmonary care, among others, have also been shown to lead to significant harm across populations. The difficulty in accessing mental health services, in particular, has led to glaringly high levels of undiagnosed depression and anxiety, contributing to a growing public health crisis across society [29, 30].

Initiatives to expand access to preventive health, which focuses on evidence‐based behavioral changes through diet, exercise, social connections, and stress management, among others, thus naturally promote quality of care at the level of the population. As importantly, they empower citizens to take charge of their health while building more substantive relationships between the community and health organizations.

Lastly, access problems can severely hinder patient engagement and trust, critical components of quality care. For instance, when patients struggle to enter the health system, communication lapses inevitably develop, which have the potential to lead to feelings of frustration and depersonalization with patients [31, 32, 33]. In this regard, access delays can contribute to a sense that the health system and/or providers are out of touch with the needs of patients, thereby negatively impacting satisfaction even before their first appointment. Because access serves to strengthen patients’ perception of control over their health and to facilitate engagement with their provider, it is a critical element of promoting patient empowerment. Intertwined with access is communication, as patients have been shown to be more accepting of delays if engagement continues to be prioritized. Thus, a consistent effort to weave communication best practices into the culture of an organization, to continually evaluate levels of access through patient surveys, and to hold teams across the organization accountable for their role in advancing communication excellence is imperative. Finally, a workforce that is oblivious to access needs can impede the development and the implementation of continuous improvement processes related to patient safety, which are critical learning systems proposed to mitigate preventable harm to patients. For instance, patient safety initiatives such as transitions of care, hand‐offs, and centralization of information exchange can be compromised when rushed, leading to a greater probability of unsafe care. In this sense, the importance of access in promoting coordinated care across teams is unquestioned.

### 4.3 Effectiveness

It has increasingly been demonstrated that access is directly correlated with health outcomes with regard to a variety of different disease conditions [34, 35, 36]. Whether the setting is heart disease, cancer, or diabetes, the timely delivery and receipt of timely care has consistently been shown to lead to improved results for patients as measured by a plethora of different endpoints. Numerous studies, for instance, have shown that delays in cancer treatment are detrimental across nearly every disease site [37, 38, 39]. A published analysis that reviewed 34 different studies including over 1 million patients showed that every 4‐week delay in treatment can increase the risk of death by 10%, with that number incrementally rising with continued lags [39].

The most conclusive data reporting on the impact of delays in access on patient care stem from the Veterans Health Administration (VHA) [40, 41, 42, 43]. According to one study on geriatric veterans, patients aged 70–74 were nearly 10% more likely to have a stroke when visiting facilities with longer waiting times [40]. An association between an increased risk of acute myocardial infarction and wait times was also established. Furthermore, at approximately 30 days of appointment wait time, older patients experienced higher mortality rates and hospitalization events. Studies from the VHA have also shown that diabetes can be prevented or managed better with timely care [41]. Penn et al. showed that as wait times have improved in recent years, there has been a parallel improvement in patient satisfaction, validating the premise that patients value access [42]. Conversely, Wong et al. showed that patients reporting longer usual wait times were significantly more likely to seek care elsewhere [43].

Some of the most striking literature on how delays in care affect outcomes arose from the COVID‐19 pandemic [44, 45, 46, 47]. Gertz et al. conducted online cross‐sectional surveys of over 300,000 subjects during the pandemic and showed that approximately 20% of respondents reported delaying healthcare. As concerningly, those patients with underlying medical conditions such as chronic lung or kidney disease were significantly more likely to forego care. Numerous studies have since shown increased mortality rates from non‐COVID causes as a result of neglected and/or delayed care [48, 49, 50].

Ultimately, studies have consistently shown a correlation between waiting times for appointments and patient satisfaction [51]. Within general healthcare, patients waiting for diagnosis, surgery, or treatment experience increases in negative affect, including anger, frustration, fear, stress, anxiety, and depression, as well as reduced self‐esteem, which worsens the longer they wait. An increasing amount of literature has also emerged specifically demonstrating the detrimental consequences for adults awaiting support for their mental health [52, 53, 54]. These studies have shown that delays in treatment were found to exacerbate existing mental and physical health symptoms.

### 4.4 Efficiency

It is important to recognize that access should not be synonymous with availability. After all, simply ensuring that every patient is able to obtain an appointment when they want one could lead to a massive amount of inefficiency and disorganization. Instead, the key aspect of successful access improvement is to ensure that the appropriate care is prioritized for those who need it most and that disparities in access are evened across populations. The focus on efficiency is thus paramount. However, this is complicated by the fact that barriers to access in healthcare are multi‐faceted, broad, and overlapping. Although commonly cited obstacles include those related to insurance coverage, affordability, social determinants, technical literacy, and/or provider availability, this list is by no means comprehensive. As importantly, more than one factor often contributes to delays in obtaining care depending on individual circumstances.

The system‐related barriers that limit access are also increasingly recognized [55, 56, 57]. Although policy makers point to a healthcare provider shortage and a workforce that is unable to keep up with the demands of a growing and aging population as the primary cause, other factors contribute as well. For instance, the use of inefficient scheduling practices, those relying on manual checks of often obsolete templates, and processes not optimizing analytics must also be considered. To optimize access, aligning supply and demand between patients and providers is critical, and many health systems are choked by inefficiency in this regard. How systems handle cancellations and “no shows” and their associated lost opportunity costs is also under scrutiny as they provide avenues for improving efficiency.

Additionally, issues related to information flow can contribute to access barriers for patients, particularly those with more complex problems [58, 59, 60, 61]. Although technology is increasingly cited as a means of decreasing inefficiencies in the healthcare system, its potential to actually lead to impediments in access must also be recognized. Given that patient visits are episodic in nature and require the sharing of information between providers, the lack of a centralized medical record system can readily contribute to poor information exchange between providers. For instance, problems with interoperability and integration with medical record storage systems can lead to increased fragmentation of visits and reduced access. The lack of compatibility between electronic health records and hospital management software can result in significant impediments in accessing patient information, thus hindering the effective coordination of care. Additionally, these barriers can lead to miscommunication with patients and payers, errors in capturing relevant information, duplicative testing, and/or missed opportunities for reimbursement [62]. It must be recognized that many patients, particularly those on the lower end of the socioeconomic spectrum, have trouble accessing digital technology, which can facilitate the scheduling of appointments, checking results, and/or communicating with providers—increasing inefficiency throughout the system [63]. For instance, underrepresented minorities have been shown to have more difficulty accessing their medical records online [64]. More recently, the term “digital redlining” was introduced to describe racialized inequities in access to technology infrastructure, including access to healthcare, education, employment, and social services [65].

### 4.5 Equity

Social determinants of health, including economic stability, education, and neighborhood conditions, can create barriers for individuals and communities, particularly marginalized groups, limiting their ability to access timely and quality care. Indeed, the influence of deeply rooted societal factors in propagating inequities in healthcare access has been well established [66]. Social determinants of health, including factors related to income, education, employment, housing, transportation, and geography, among others, have been shown to contribute significantly to access by creating external obstacles unique to vulnerable segments [66, 67, 68]. For instance, patients from impoverished backgrounds not only struggle more with insurance coverage but also face challenges with respect to taking time off work, arranging for family care needs, and/or coordinating transportation. Studies have also shown that higher quality medical services generally are located in more affluent communities, thus creating de facto “healthcare deserts” [69, 70, 71]. Indeed, statistics from the American Hospital Association estimate that approximately 3.5 million patients go without care because they cannot access transportation to their providers [72]. This access dilemma is particularly problematic for patients residing in rural communities where the geographical distance to healthcare centers can be vast. The role of education is also impactful given that it allows patients to have a better awareness of their health conditions and to thus prioritize access. Unsurprisingly, a plethora of evidence exists demonstrating the strong association between educational attainment and mortality from many diseases [73, 74, 75]. Similarly, while wages and income are not health outcomes per se, they are closely linked with health outcomes because they provide access to health‐related resources, such as food, a safe environment, and medical services [76].

The role of discrimination in contributing to access inequities must also be recognized. Treatment bias based on race, immigration status, sex, gender, disability, and sexual orientation has been shown to create less than welcoming environments, thus leading some members of these protected classes to postpone or forgo care altogether [77]. For instance, the perception that Black patients have naturally higher thresholds for pain compared to White patients likely contributed to the former experiencing longer waits and access delays in conjunction with their complaints not being taken seriously [78]. The view that the lives of immigrant and/or non‐English‐speaking patients might not matter as much as native‐born individuals also can create access barriers in a similar fashion. As such, the influence of implicit bias in sowing distrust among underserved groups must be acknowledged. Given that patients from disadvantaged backgrounds have reported more negative interactions with the healthcare system and are more likely to perceive their experiences as “cold, unfriendly, and insensitive,” factors related to comfort level can drive barriers to access [79]. In these cases, patients who feel that the healthcare system only caters to the privileged might fall through the cracks due to a perceived indifference for their beliefs and unique backgrounds. For instance, medical appointments can conjure up emotions of fear and anxiety that can be exacerbated in certain underserved communities even accounting for intergenerational trauma [80]. How social determinants and class factors impact how populations perceive the friendliness of healthcare systems and quality of care warrants further investigation.

Structural factors also can contribute to barriers to access. For instance, historical policies like redlining (to designate “desirable” and “undesirable” neighborhoods based largely on income) have led to both racial segregation and disparities in access to resources and services, like high‐quality hospitals [81]. The implications on healthcare access and quality have been shown to be profound. Indeed, studies have shown that historical redlining is linked to increased risk of diabetes, hypertension, cardiovascular disease, and early mortality, among other conditions [82, 83, 84]. Eberth et al. examined the correlation between geography and socioeconomic variables in the potential utilization of health services across the country. Using data from the 2019 American Hospital Association annual survey, they identified significant disparities among minority populations based on the measured distance to the nearest hospital offering emergency services, trauma care, obstetrics, outpatient surgery, intensive care, and cardiac care [85]. Appolon et al. similarly demonstrated the impact of redlining on pharmacy access [86]. Notably, the negative consequences were particularly pronounced in most socioeconomically deprived neighborhoods, where redlining was associated with 35% and 51% decreased odds of living within 1 and 2 miles of a pharmacy, respectively. Finally, the rate of uninsurance—which can directly impact access to healthcare and healthcare affordability—was much higher in formerly redlined districts, with 18% of adults in those areas saying they don't have payer coverage compared to 6% of those living in A‐rated census tracts [87].

### 4.6 Patient‐Centeredness

Although patient‐centered care is commonly touted as a goal of the healthcare system, the term has taken on various meanings. At its core, patient‐centered care refers to the ability to address not only a patient's medical condition but also, as importantly, the associated emotional, mental, spiritual, social, and financial considerations. Recognizing that priorities vary from individual to individual is the initial step in providing patient‐centered care. Although studies have consistently shown that access is a factor that is prioritized by all patients, it is clear that some subjectivity is inherent in its definition [88].

Ultimately, patient‐led definitions are essential in order to set objectives to better understand that relationship between access and patient‐centered care. Levesque et al. established a well‐known conceptual framework to outline the dimensions that relate to patients navigating the continuum of the healthcare environment [89]. The authors proposed a foundation integrating five dimensions of accessibility: approachability, acceptability; affordability, appropriateness, and availability and accommodation. These were superimposed on five corresponding factors that were patient‐centric (ability to perceive, ability to seek, ability to reach, ability to pay, ability to engage). Using this model, the researchers suggested that the concept of access could be synthesized to improve care across populations.

Kurpas et al. convened focus groups to gather qualitative data on perspectives of access for older adults [90]. Notable findings were that participants described a lack of integration between health and social care systems with differing priorities and disconnected budgets. Among the biggest complaints were those related to a low‐functioning and uncoordinated social system leading to bureaucratic hassles, which were the source of delays in care. Oliver et al. performed a cost–utility analysis to examine trade‐offs that patients might consider during appointment bookings in two urban family medicine clinics [91]. Using six different simulated clinical scenarios across a number of key access and continuity attributes, they showed that patients preferred timely access to their primary care team over other attributes in the majority of cases. However, Gerard et al. showed that other factors contribute to patients’ definition of access [92]. For instance, having the ability to see a specialist of choice and to maintain a relationship with a preferred provider were also influential to patients. The investigators also found that preferences varied by gender, employment, and career status, again demonstrating that defining “access” isn't quite as straightforward as it seems. Notably, the Office of Disease Prevention and Health Promotion proposed four broad objectives: increasing the proportion of people with health insurance, the proportion of people with dental insurance, the proportion of adults who receive recommended evidence‐based preventive healthcare, and the proportion of adolescents who obtain preventive healthcare visits [93].

From a practical standpoint, the concept of care‐related financial toxicity is increasingly being recognized as a burden for patients [94]. As such, patient‐centered care, although generally touted to refer to the overall patient experience, must also consider financial expenses. Notably, in many countries such as the United States, access is hindered for the significant proportion of the public that continues to lack health insurance [95]. However, simply having insurance does not necessarily mean that access is guaranteed, as logistical and bureaucratic factors can serve as a major deterrent for patients. Additionally, studies have shown that prior authorization, which negatively impacts the ability of patients to see specialists, obtain diagnostic studies, acquire medications, and/or even start treatment, can lead to major delays in care [96, 97, 98]. In one survey of physician attitudes, greater than 50% of the respondents acknowledged choosing inferior treatments for their patients at least weekly because of the perceived prior authorization burden for preferred agents [99]. Furthermore, half of the respondents reported personally having a patient who experienced serious adverse events due to prior authorization‐related care delays. Another survey conducted by the American Medical Association showed that more than half of physicians reported that prior authorization has “impacted patient job performance” [100]. With respect to access, a staggering 94% of physicians stated that “delays in care” resulted from prior authorization. As concerningly, more than three‐quarters of the physicians reported that “treatment abandonment” occurred because of prior authorization, and more than one‐third reported that prior authorization led to a serious adverse event including hospitalization, disability, and/or death for a patient while waiting for care.

The escalating cost of healthcare for patients, particularly high out‐of‐pocket expenses, is another well‐documented access barrier. Data from a West Health and Gallup poll found that 29% of adults reported putting off medical treatment because of out‐of‐pocket costs between 2001 and 2021 [101]. Although 34% of Americans with an annual household income of less than $40,000 were likely to skip or delay healthcare for a serious medical condition, a considerable proportion of higher earners (18%), defined as those with household income greater than $100,000, also stated the same. According to 2023 data from the Commonwealth Fund, the United States has the starkest income‐based health disparities compared to other similarly developed nations [102]. Indeed, 46% and 27% of American adults have skipped a medical visit, test, treatment, follow‐up, or prescription fill within the last year solely because of cost among low‐income and high‐income earners, respectively. Campbell et al. evaluated the influence of financial barriers on health outcomes in a cohort of 120,752 patients with cardiovascular‐related chronic disease, including hypertension, diabetes, heart disease, or stroke. Notably, they showed that patients with financial barriers had inferior outcomes with higher rates of disease‐related hospitalization and mortality, even after adjusting for potential confounding variables such as age, sex, education, comorbidity, and smoking status, compared to those without financial barriers [103].

## 5 Conclusion

The relationship between access and quality is increasingly being explored, and future research in this area will likely center around the six domains of healthcare quality as reviewed. Given how the concept of access is being continually refined, the effects of societal, government, and system‐based initiatives will need to be monitored to ensure they are keeping up with evolving definitions. Indeed, the widespread adoption of digital health strategies into practice has introduced many new perspectives with access. For instance, digital communication tools, such as mobile health apps, telemedicine, and online health information resources, including live chatbots, offer unique opportunities to reach a wide range of demographics, regardless of their geographic location, socioeconomic status, or educational background. Innovative open access scheduling systems utilizing online self‐service applications have also been proposed as a means to streamline the appointment process—so patients can schedule, reschedule, or cancel appointments whenever it is convenient for them, which is often outside provider office hours [104, 105, 106]. The popularity of same‐day and expanded access—which allows patients to be seen within hours from scheduling an appointment and at times that might be more convenient for the working population, respectively—has also been increasingly demonstrated [107]. Studies evaluating the role of information exchange and data operability between sites will thus be useful to optimize access in these scenarios. Similarly, attention to ensuring that digital literacy is evenly maintained across society is necessary so that segments of the population are not left underserved.

Lastly, the design of value‐based initiatives to address social determinants to promote health equity will be paramount. Models under the Center for Medicare and Medicaid delivery system are increasingly surveying for gaps with respect to social needs and implementing community‐based preventive programs [108]. Recently, numerous states required Medicaid managed care plans to screen for and/or provide referrals for social needs, and a recent survey found that nearly all responding plans reported activities to address social determinants of health [109]. Along these lines are efforts to expand coverage for the underserved segments of the population, particularly with respect to preventive care [110]. Studies promoting health literacy to bridge the increasing “digital divide” that exists between the educated and uneducated are also needed [111]. Furthermore, innovative programs to promote price transparency in a standardized way such that financial toxicity can be mitigated also have the potential to improve access [112].

Achieving alignment in every phase of access improvement is imperative and requires concerted efforts to be inclusive, pro‐active, and visible. Only through adherence to these standards—combined with a committed, thoughtful approach to execution and innovation—will positive change truly occur. Ultimately, access improvement will not happen overnight nor be straightforward. Instead, the thoughtful and concerted engagement of all stakeholders, including patients, providers, health systems, insurance companies, and legislative bodies, among others, will be necessary to lead to meaningful gains. Given the evolving relationship between access and quality, ongoing research evaluating the effectiveness of patient‐centered strategies to improve care delivery will be critical to refine this paradigm in the future.

## Author Contributions

**Allen M. Chen**: conceptualization, investigation, writing – original draft, writing – review and editing, visualization, validation, methodology, software, project administration, formal analysis, supervision, resources, data curation.

## Disclosure

The author has nothing to report.

## Ethics Statement

No human subjects were involved in this work.

## Conflicts of Interest

The author declares no conflicts of interest.

## Data Availability

There are no original data arising from this work.
